# Exploring virtual reality gaming‐related cybersickness in Alzheimer’s disease

**DOI:** 10.1002/alz.083889

**Published:** 2025-01-09

**Authors:** Rashmita Chatterjee, Zahra Moussavi

**Affiliations:** ^1^ University of Manitoba, Winnipeg, MB Canada

## Abstract

**Background:**

In the last decade, virtual reality has become a popular tool for rehabilitation. It is quite useful in spatial rehabilitation for Alzheimer’s disease (AD) as it allows safe navigation in a virtual environment which looks realistic. However, a drawback of virtual reality is cybersickness. The symptoms and severities of cybersickness can vary among users. Possible cybersickness symptoms include headache, nausea, disorientation.

Cybersickness can be distracting for participants and can affect their performance on the virtual reality task. Hence, in our rehabilitation game called Barn Ruins Navigation (BRN), we used software design mechanisms that would reduce cybersickness, including (1) keeping the field of view to 60 degrees, (2) lowering speed of rotation and translation, (3) using a joystick which causes less cybersickness, and (4) using a laptop screen instead of a head‐mounted display.

There have been contradicting results on the effects cybersickness has on people with AD. We wanted to test if cybersickness felt by the BRN game differed between people with AD and cognitively healthy adults.

**Method:**

Cybersickness was measured using the Simulator Sickness Questionnaire (SSQ) after playing BRN. Thirty adults were recruited for the BRN validation study between the ages of 20‐88. This includes ten younger adults (five males, 26±3.39 years), ten cognitively healthy older adults (three males, 70.7±5.31 years), and ten people living with mild to moderate AD (seven males, 77.8±5.94 years). All participants played the game once.

**Result:**

Regression analysis on the data showed that males had significantly lower SSQ scores by about 17.47 points compared to females (p = 0.0106), while adjusting for participant groups. The AD group had the highest SSQ scores in comparison to cognitively healthy older adults and younger adults while adjusting for sex. Only the younger adults had a statistically lower SSQ score than the AD group by 18 points (p = 0.023).

**Conclusion:**

We found males were less prone to cybersickness than females, matching the cybersickness literature. Our study found people with AD were more prone to cybersickness, however not to a statistically higher degree than cognitively healthy older adults. We would need a larger sample size to draw solid conclusions.

